# Procedure‐related readmissions following endoscopic retrograde cholangiopancreatography in a liver transplant cohort

**DOI:** 10.1002/jgh3.70008

**Published:** 2024-10-03

**Authors:** Jennifer Gu, Leonardo Zorron Cheng Tao Pu, Jonathan Ng, Kim H Be, Rhys Vaughan, Sujievvan Chandran, Marios Efthymiou

**Affiliations:** ^1^ Medicine, Dentistry and Health Sciences University of Melbourne Parkville Victoria Australia; ^2^ Department of Gastroenterology and Hepatology Austin Health Melbourne Victoria Australia; ^3^ Department of Medicine Monash University, Peninsula Health Campus Frankston Victoria Australia

**Keywords:** adverse events, complications, endoscopic retrograde cholangiopancreatography, liver transplantation, readmissions

## Abstract

**Background and Aim:**

Data on post‐endoscopic retrograde cholangiopancreatography (ERCP) adverse events and readmission rates in liver transplantation (LT) patients remain scarce. This study determined the 30‐day procedure‐related readmission rate following ERCP in an LT cohort at an Australian tertiary academic center.

**Methods:**

All unplanned readmissions within 30 days following ERCP in orthotopic LT patients between December 2012 and August 2021 were retrospectively identified. Demographic data, procedure variables, and readmission characteristics were also collected.

**Results:**

Forty‐five procedure‐related readmissions were identified (3.3%) from a total of 1369 ERCP procedures. This included 33 cases of cholangitis (2.4%), 7 cases of nonspecific abdominal pain (0.5%), 5 cases of mild post‐ERCP pancreatitis (0.5%), and 3 cases of bleeding (0.2%). No procedure‐related mortality was observed.

**Conclusion:**

The procedure‐related readmission rate following ERCP in this LT cohort was 3.3%, which is likely lower than comparable studies carried out on the overall population.

## Graphical abstract

This study presents the first Australian data on procedure‐related readmissions following endoscopic retrograde cholangiopancreatography (ERCP) in a liver transplant cohort. Study results suggest procedure‐related readmissions within 30 days after ERCP were likely lower than comparable studies carried out on the overall population.

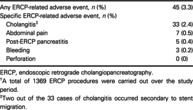

## Introduction

Liver transplantation (LT) is a life‐saving treatment for patients with fulminant liver failure and end‐stage liver disease with excellent long‐term survival rates. However, post‐transplant biliary adverse events such as biliary strictures, leaks, and stones represent a significant source of morbidity in 10–30% of these patients, with higher rates observed in living donor LT recipients. Timely and appropriate management of these adverse events is critical to avoid the need for re‐transplantation.^1^, ^2^

**Figure 1:**
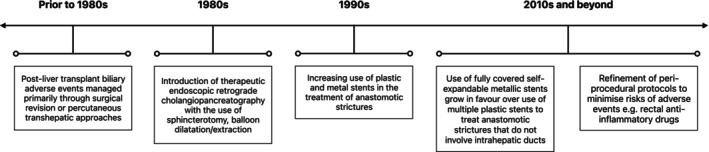
Trends of endoscopic retrograde cholangiopancreatography in the management of post‐liver transplant biliary complications.^3^, ^4^, ^5^, ^6^, ^7^, ^8^, ^9^

Treatment for post‐transplant biliary adverse events has evolved considerably over the years, as depicted in Figure 1.^3^, ^4^, ^5^, ^6^, ^7^, ^8^, ^9^ In recent decades, endoscopic retrograde cholangiopancreatography (ERCP) has become a commonly used therapy for managing these adverse events, and is considered the initial treatment of choice for biliary strictures in LT recipients as indicated by the American Society for Gastrointestinal Endoscopy (ASGE) and European Society of Gastrointestinal Endoscopy guidelines.^1^, ^5^, ^10^ Both anastomotic and nonanastomotic biliary strictures are key indications for ERCP in these patients and are typically managed with serial ERCP procedures involving insertion of single or multiple stents with or without balloon dilatation, with plastic stents requiring three‐monthly replacements over the span of 12 months.^5^ As such, ERCP is a commonly performed procedure in LT patients. Although there is a large body of evidence to suggest the safety of ERCP in the general population, there are limited data regarding the rate of adverse events in the LT cohort. The purported incidence of post‐ERCP adverse events ranges vastly from 0.7 up to 22.7% (Table 1) with no consensus on how this compares to the general population.^11^, ^12^, ^13^, ^14^, ^15^, ^16^, ^17^, ^18^, ^19^, ^20^, ^21^, ^22^, ^23^, ^24^, ^25^, ^26^, ^27^, ^28^, ^29^, ^30^

**Table 1:** Post‐endoscopic retrograde cholangiopancreatography (ERCP) adverse events in liver transplant patients—A review of the literature^†^

Author	Location	Number of ERCP^‡^	Adverse event rate %^§^
Primary outcome: Post‐ERCP complications			
Ramesh 2014^11^	UK	210	2.86%
Thiruvengadam 2020^12^	USA	937	5.12%
Husing 2015^13^	Germany	454	5.29%
Ambrus 2015^14^	Denmark	292	8.22%
Balderramo 2011^15^	Spain	243	9.05%
Primary outcome: ERCP treatment success or failure			
Buxbaum 2011^16^	USA	715	0.70%
Eminler 2017^17^	Turkey	148	3.38%
Egea 2019^18^	Spain	168	6.55%
Heinemann 2019^19^	Germany	586	6.83%
Ronning 2019^20^	Sweden	418	7.66%
Eslami 2022^21^	Iran	137	8.76%
Sanna 2011^22^	Italy	150	10.00%
Martins 2015^23^	Brazil	341	12.61%
Primary outcome: Efficacy of stent type or stenting protocol			
Barakat 2018^24^	USA	410	1.71%
Cantu 2019^25^	Italy	656	7.77%
Sandru 2022^26^	Romania	106	8.49%
Martins 2018^27^	Brazil	201	12.44%
Poley 2013^28^	Netherlands	155	14.19%
Hsieh 2013^29^	USA	187	18.72%
Ahmed 2022^30^	UK	176	22.73%

^†^ A comprehensive review of the literature was carried out with the following exclusion criteria: studies with n (ERCP procedure) >100, studies published prior to 2011, studies where total number of ERCP procedures and/or actual total number of independent complications were omitted.

^‡^ Performed in liver transplant patients only.

^§^ Where necessary, complication rates were manually calculated based on available data, for example, when adverse event rates were presented separately for different study groups, when clinically insignificant events were included in overall adverse event rate or when a total adverse event rate was not provided. Any complications that lacked clarity in their clinical significance were not included when calculating the adverse event rate.

On a broader scale, hospital readmission rate is a metric thought to reflect hospital quality of care. The procedure‐related readmission rate is also a useful surrogate for post‐procedural adverse event rate. Recent studies found that in mixed cohorts of non‐LT and LT patients, procedure‐related readmissions occurred at a rate of 1.4–10.2% in the 30 days following an ERCP procedure (calculated manually based on available data).^31^, ^32^, ^33^ To our knowledge, there are no published data on LT patients alone.

This retrospective study aimed to determine the incidence of unplanned readmissions associated with procedure‐related adverse events within 30 days after ERCP, in an LT cohort at a single Australian center.

## Methods

### Patients

**Figure 2:**
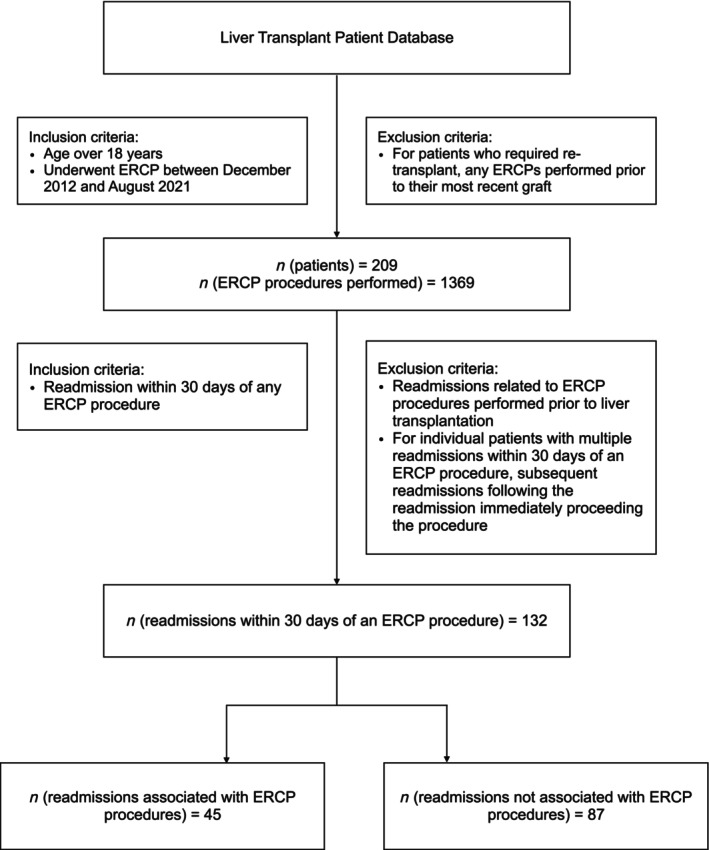
Study flow chart. Flow diagram for initial search of database, excluded patients, endoscopic retrograde cholangiopancreatography procedures and readmissions and final readmissions included in study analysis.

Patients (>18 years) who underwent ERCP following orthotopic LT at the Austin Hospital in Melbourne, Australia, were retrospectively identified through a LT database. Three endoscopists with over 2000 cases of experience (ME, SC, RV) performed the ERCP procedures. As this is a training hospital, an advanced endoscopy fellow was also involved in most cases. All unplanned readmissions occurring within 30 days of the procedure between December 2012 and August 2021 were included. Inclusion and exclusion criteria for patients and readmissions are described in Figure 2. Ethics approval for this study was granted by the Austin Health Ethics Committee.

### Data collection and statistical analysis

Electronic medical records of eligible patients were retrospectively reviewed. Demographic data, ERCP procedure characteristics, and indication for ERCP were extracted. The characteristics of the readmission were also examined as well as occurrence of the following within the 30 days following the procedure: post‐ERCP pancreatitis (PEP), cholangitis, abdominal pain, bleeding, perforation, procedural‐related mortality, and all‐cause mortality. Descriptive statistics were calculated using Microsoft Excel.

### Defining post‐ERCP adverse events

Pancreatitis was defined as per the Atlanta criteria requiring “two of the following three features (i): abdominal pain consistent with acute pancreatitis; (ii) serum lipase activity (or amylase activity) at least three times greater than the upper limit of normal; and (iii) characteristic findings of acute pancreatitis on imaging.”^34^ Any occurrences of pancreatitis fulfilling these criteria and occurring within 14 days after ERCP were included. Cholangitis was defined following the 2018 Tokyo Guidelines, the criteria for which can be found in the Appendix I.^35^ Abdominal pain was defined as any nonspecific abdominal pain that could not be attributed to any other known cause such as PEP or perforation. Bleeding was defined as clinical evidence of bleeding, hematemesis and/or melena, or a drop in hemoglobin by 2 g/L without other cause. Perforation was defined by evidence of gas or luminal content outside the gastrointestinal tract as determined by imaging.^36^

## Results

### Patients

Over the study period, 209 patients with a transplanted liver in situ underwent an ERCP procedure, with a total of 1369 procedures performed. Of the 209 patients, 103 were readmitted to hospital within 30 days of their ERCP procedure, 15 of whom had multiple readmissions. This culminated in a total of 132 readmissions. The basic demographic data for these readmissions are reported in Table 2. The median age of patients at the time of ERCP was 59 years. The most common primary indication for LT was Hepatitis C (37.9%). All transplanted livers were from deceased donors with 121 receiving whole livers (91.7%) and 11 receiving right lobe split livers (8.3%). The median time between transplant and ERCP was 195 days or approximately 6.5 months (range: 7–7257 days).

**Table 2:** Demographic data of readmissions and ERCP procedure characteristics (*n* = 132^†^)

Age at the time of ERCP in years, median [IQR]	59 [53 to 63]
Gender, *n* (%)	
Male	82 (62.1)
Female	50 (37.9)
Transplant indication, *n* (%)	
Hepatitis C	50 (37.9)
Non‐alcoholic steatohepatitis	19 (14.4)
Autoimmune hepatitis	14 (10.7)
Alcoholic cirrhosis	12 (9.1)
Hepatitis B	12 (9.1)
Primary sclerosing cholangitis	7 (5.4)
Cryptogenic cirrhosis	5 (3.8)
Primary biliary cirrhosis	4 (3.1)
Acute liver failure	3 (2.3)
Other^‡^	6 (4.6)
Transplant anatomy, *n* (%)	
Whole liver	121 (91.7)
Right lobe split liver	11 (8.3)
Time between transplant and ERCP in days, median [IQR]	195 [82.75 to 810.75]

ERCP, endoscopic retrograde cholangiopancreatography; LT, liver transplantation.

^†^ Over the study period, there were a total of 132 readmissions involving 103 LT patients, 15 of whom had multiple readmissions. The demographic data were based on the 132 readmissions, rather than unique patients as the encounters were independent to each other with its own associated risk.

^‡^ Other transplant indications include: Biliary Atresia, Polycystic Kidney Disease, Hereditary Hemorrhagic Telangiectasia, Acute Budd Chiari Syndrome, and two cases of multifactorial liver disease—one with Hemochromatosis, Alcoholic and methotrexate toxicity, and one with Alpha 1 Anti‐Trypsin Deficiency and Alcoholic cirrhosis.

### ERCP procedure variables

Table 3 presents ERCP procedure‐related variables. Sixty ERCP procedures were performed for management of anastomotic stricture (45.5%) and four were performed for management of nonanastomotic strictures (3.0%). Other indications included abnormal or worsening LFTs (22.0%), bile leak (11.4%), cholangitis (9.8%), stones or cast on imaging (6.8%), or miscellaneous indications such as T tube removal and suspected biloma (1.5%). Antibiotic prophylaxis was administered to patients in 123 of the ERCP procedures (93.2%). Rectal indomethacin was given in 19 cases for PEP prophylaxis (14.4%). A sphincterotomy was performed in 47 cases (35.6%). The pancreatic duct (PD) was cannulated in 13 cases (9.8%), with a stent prophylactically inserted in 9 cases (6.8%). A biliary stent was inserted in 98 cases (74.2%), with 92 cases involving plastic stents, 4 cases involving Kaffes intraductal metal stents (Niti‐S Kaffes, Taewoong Medical, South Korea), and 2 utilizing self‐expandable metallic stents. Dilation of the bile duct for both anastomotic and nonanastomotic strictures was performed in 42.4% of all cases. This was typically done using a Hurricane RX Biliary Balloon Catheter (Boston Scientific) over a wire, most commonly 4–6 mm in diameter.

**Table 3:** Endoscopic retrograde cholangiopancreatography (ERCP) procedure characteristics (*n* = 132)

Indication for ERCP, *n* (%)	
Anastomotic stricture	60 (45.5)
Abnormal or worsening LFTs	29 (22.0)
Bile leak	15 (11.4)
Cholangitis	13 (9.8)
Stones or cast	9 (6.8)
Non‐anastomotic stricture	4 (3.0)
Other^†^	2 (1.5)
Interventions used, *n* (%)	
Antibiotic prophylaxis	123 (93.2)
Rectal indomethacin	19 (14.4)
Sphincterotomy	47 (35.6)
Sphincteroplasty	1 (0.8)
Common bile duct cannulation	129 (97.7)
Balloon or basket dredging	83 (62.9)
Pancreatic duct cannulation	13 (9.8)
Pancreatic duct injection	2 (1.5)
Pancreatic duct stent	9 (6.8)
Dilation of bile duct—anastomotic stricture	44 (33.3)
Dilation of bile duct—non‐anastomotic stricture	12 (9.1)
Any biliary stent used, *n* (%)	98 (74.2)
Type of biliary stent (*n* = 98), *n* (%)	
Plastic	92 (93.9)
Kaffes intraductal stent	4 (4.1)
SEMS	2 (2.0)
Number of biliary stent(s) if plastic stent(s) (*n* = 92), *n* (%)	
1	75 (81.5)
2	10 (10.9)
3	6 (6.5)
4	0 (0)
5	1 (1.1)
Cholangioscopy, *n* (%)	3 (2.3)
Cholangioscopy indication (*n* = 3), *n* (%)	
Common bile duct stone	2 (66.7)
Stricture cannulation	1 (33.3)

LFTs, liver function tests; SEMS, self‐expandable metallic stent.

^†^ Other indications for ERCP include: T tube removal and suspected collection/biloma.

### Readmissions

Out of a total of 1369 ERCP procedures, this study identified 132 readmissions occurring within 30 days of an ERCP, 45 of which were associated with an ERCP‐related adverse event (3.3%). These are reported in Table 4. The most frequently occurring adverse event was cholangitis, of which there were 33 cases (2.4%), 2 of which occurred secondary to stent migration. There were also seven cases of abdominal pain (0.5%), five cases of PEP, all of which were mild (0.4%), and three cases of bleeding (0.2%). One case of mortality was noted, occurring secondary to progression of an underlying malignant disease process and was unrelated to the preceding ERCP. There were no cases of perforation, cardiopulmonary events, or procedure‐related mortality. The median time between ERCP and readmission was 10 days (interquartile range: 5–19) and median length of stay was 4 days (interquartile range: 2–7.25).

**Table 4:** ERCP‐related readmissions (*n* = 1369^†^)

Any ERCP‐related adverse event, *n* (%)	45 (3.3)
Specific ERCP‐related adverse event, *n* (%)	
Cholangitis^‡^	33 (2.4)
Abdominal pain	7 (0.5)
Post‐ERCP pancreatitis	5 (0.4)
Bleeding	3 (0.2)
Perforation	0 (0)

ERCP, endoscopic retrograde cholangiopancreatography.

^†^ A total of 1369 ERCP procedures were carried out over the study period.

^‡^ Two out of the 33 cases of cholangitis occurred secondary to stent migration.

## Discussion

In this retrospective study, the incidence of 30‐day procedure‐related readmissions following ERCP in our LT cohort was 3.3%. To our knowledge, there are only three studies that have assessed procedure‐related readmission rates in mixed cohorts of both non‐LT and LT patients.^31^, ^32^, ^33^ Their findings indicate a range of 1.4–10.2%, with the lowest figure coming from a single‐center study. This single‐center study also found history of LT to be predictive of a readmission; however, the analysis did not discriminate between procedure‐related and all‐cause readmissions.^32^ The other studies examined data from either a US nationwide database^31^ or from three different US states^33^ and both revealed a higher incidence of ERCP‐attributed readmissions in comparison with our results. As such, it is possible that our findings in an LT cohort reflect a lower procedure‐related readmission rate compared with mixed cohorts. To our knowledge, there are no other published data on procedure‐related readmission rates following ERCP in LT patients alone.

In this study, we elected to use the 2018 Tokyo Guidelines to define post‐ERCP cholangitis.^35^ According to other commonly used guidelines,^36^, ^37^ patients must have a temperature of greater than 38°C for more than 24 h with cholestasis to qualify for a diagnosis of post‐ERCP cholangitis. However, many patients in this transplant cohort with a clinical diagnosis of cholangitis remained afebrile, likely due to their immunosuppressed state and reduced ability to mount a robust inflammatory response. The 2018 Tokyo Guidelines were able to capture these cases of cholangitis, as evidence of systemic inflammation is not compulsory for a diagnosis.^35^ (see Appendix I, Table A1 for full diagnostic criteria). This highlights one of the challenges in classifying adverse events in endoscopic procedures for which there is no current consensus. Using the 2018 guidelines, the incidence of cholangitis‐related readmissions was found to be 2.4% in our study. This was the most frequently occurring adverse event.

This study also found five cases of mild PEP (0.4%). International guidelines recommend several measures to reduce the risk of PEP including administration of rectal nonsteroidal anti‐inflammatory drugs, prophylactic PD stenting in high‐risk patients, as well as periprocedural intravenous hydration.^38^, ^39^, ^40^ Rectal indomethacin was administered in one of the 5 PEP cases in this study. Overall, 14.4% of all patients received rectal indomethacin. This may reflect the 10‐year time span of this study and the relatively recent introduction of rectal indomethacin into our standard protocol. Indomethacin is also only routinely given in native papilla ERCP procedures. Most of this patient cohort have had multiple ERCP procedures as part of a stenting protocol and therefore had already undergone sphincterotomy, which is typically only performed at the index ERCP.

Difficult access of the biliary duct and PD cannulation are both risk factors for PEP.^38^ Two of the 5 PEP cases in this study were performed on native papillae and involved difficult cannulation of the biliary duct. Prophylactic PD stent insertion following inadvertent PD cannulation was performed in both cases. Most cases of PD cannulation were followed by prophylactic PD stent insertion (9 of 13), with only two resulting in PEP‐related readmission. Data regarding periprocedural intravenous hydration were not collected; however, this is part of standard practice.

Previous studies have postulated that the immunosuppressed state of transplant patients may reduce the risk of PEP. Two recent studies suggest that tacrolimus at a certain level significantly reduces the chance of PEP, with indomethacin having an additive effect.^12^, ^41^ Although our study found a low rate of PEP during readmission, it is unclear whether our results support this theory as we did not examine the absolute adverse event rate. Nevertheless, Radadiya et al. found the rate of pancreatitis‐associated readmission to be 7.3%, which is higher than our results.^31^ Krill et al. observed a 0.20% rate of PEP‐related readmissions; however, the overall adverse event rate was found to be higher at 3.2%.^32^ Beyond the established role of rectal indomethacin, the potential efficacy of immunosuppressive agents for PEP prophylaxis is an area that requires further study.

There were no other severe adverse events associated with readmissions in this study. In the seven readmissions involving nonspecific abdominal pain, lipase was normal. The pain was either self‐limited or relieved with simple analgesia and all patients were discharged within 3 days of admission. In all three cases of bleeding requiring readmission, sphincterotomy was performed.

No cases of procedure‐related mortality occurring during a readmission were identified in this study. There was one case of mortality in which the primary cause of death was identified as progression of an underlying malignant disease process, unrelated to the preceding ERCP procedure.

Although this study suggests a relatively low rate of readmission associated with ERCP adverse events, such readmissions still contribute greatly to post‐transplant morbidity and lead to significant costs incurred for hospitals and the wider healthcare system. When compared with the general population, liver transplant patients have been found to be at a higher risk of adverse events following sphincterotomy.^42^ Post LT index ERCP^43^ and time since LT within 90 days^14^ have also been found to be risk factors for developing adverse events. Conversely, as previously noted, earlier studies have also identified possible protective factors in this cohort of patients such as the use of immunosuppressive therapies such as tacrolimus and steroids, which may protect against developing post‐ERCP adverse pancreatitis.^12^, ^41^, ^43^ Future studies should further investigate both protective and predisposing factors contributing to adverse events and readmissions following ERCP in LT recipients, with pertinent results being applied to the general population.

As data were only collected for ERCP procedures leading to readmission, carrying out a meaningful univariate or multivariate analysis was not possible in this retrospective study. The other limitation of this study arises from the primary outcome being ERCP‐related readmission rate rather than overall adverse event rate. Adverse events occurring during the same admission as the procedure and undocumented events due to presentation to a different facility or mild symptoms not requiring admission were not captured. Therefore, direct comparisons with studies examining adverse event rates cannot be made as this study may underreport the true number of adverse events.

Nevertheless, this study examined the topic of procedure‐associated readmissions, which raises important questions around post‐transplant morbidity and the significant costs associated with hospital readmission. The incidence of procedure‐related readmissions within 30 days of ERCP in our LT cohort was determined to be 3.3%, which is likely lower than comparable studies carried out on the overall population.

## Appendix I

### I.1

**Table A1:** Tokyo guidelines 2018 diagnostic criteria^35^

Systemic inflammation A‐1. Fever and/or shaking chills A‐2. Laboratory data: evidence of inflammatory response Cholestasis B‐1. Jaundice B‐2. Laboratory data: abnormal liver function tests Imaging C‐1. Biliary dilatation C‐2. Evidence of etiology on imaging (stricture, stone, stent etc.)
Suspected diagnosis: one item in A + one item in either B or C Definite diagnosis: one item in A, one item in B and one item in C
